# [Corrigendum] Inhibition of microRNA-221-5p induces osteogenic differentiation by directly targeting smad3 in myeloma bone disease mesenchymal stem cells

**DOI:** 10.3892/ol.2026.15533

**Published:** 2026-03-17

**Authors:** Fang-Yi Fan, Rui Deng, Si-Han Lai, Qin Wen, Yunjing Zeng, Lei Gao, Yao Liu, Peiyan Kong, Jiangfan Zhong, Yi Su, Xi Zhang

Oncol Lett 18: 6536–6544, 2019; DOI: 10.3892/ol.2019.10992

Following the publication of the above article, an interested reader drew to the authors’ attention that, for the osteogenic differentiation experiments shown in Fig. 4C on p. 6542, the ‘inhibitor+lv-smad3’ and ‘miR-221-5p inhibitor’ data panels appeared to contain an overlapping section of data, such that these data panels were apparently derived from the same original source where the results of different experiments were intended to have been portrayed.

The authors were able to consult their original data, and realized that this figure had inadvertently been assembled incorrectly. The revised version of Fig. 4, now showing the correct data for the ‘inhibitor+lv-smad3’ panel, is shown on the next page. The authors regret that this error occurred in the originally published version of this figure, although this did not grossly affect either the results or the conclusions reported in this article. All the authors agree with the publication of this Corrigendum, and thank the Editor of *Oncology Letters* for allowing them the opportunity to publish this; furthermore, they apologize to the readership for any inconvenience caused.

## Figures and Tables

**Figure 4.**
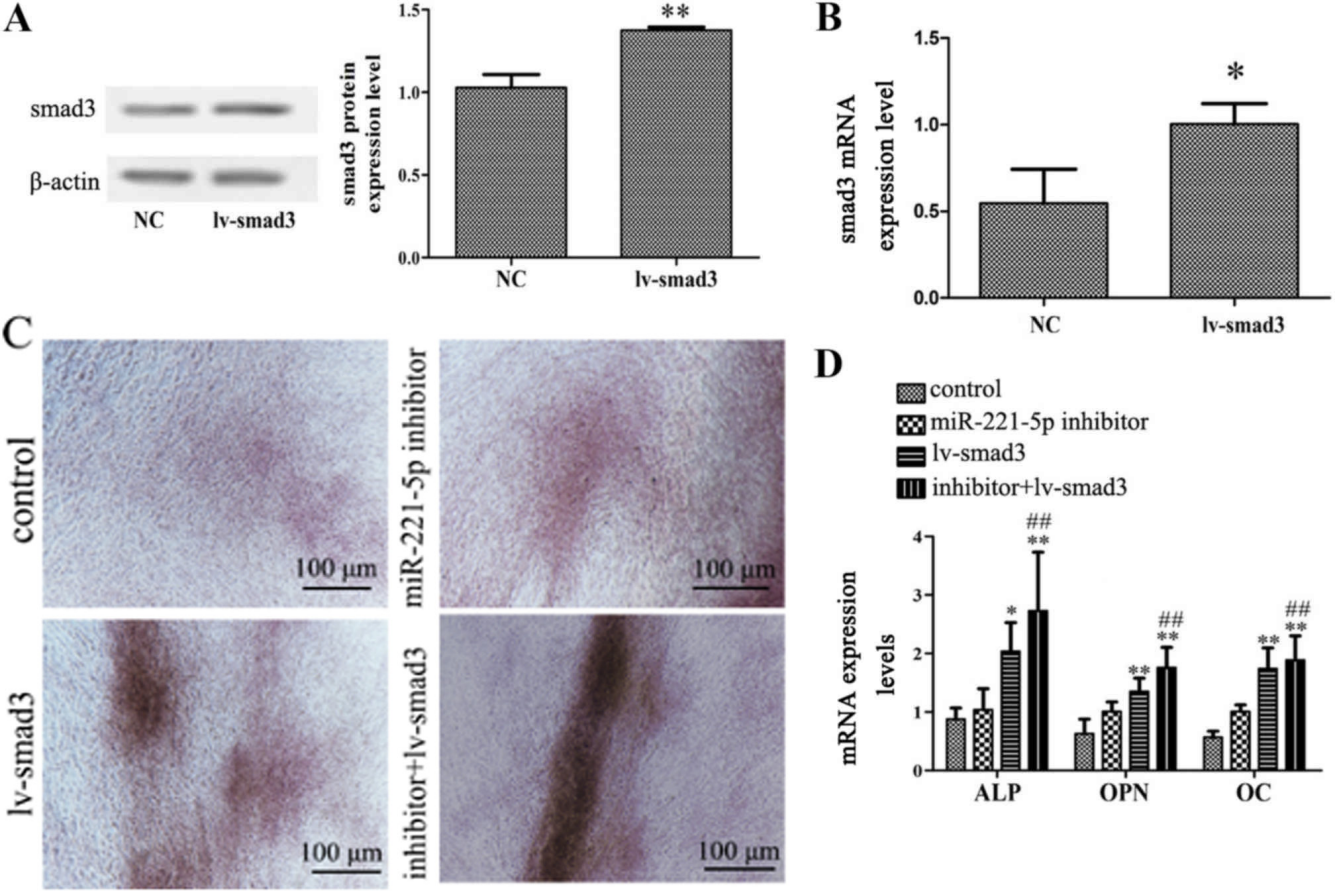
Effects of smad3 overexpression on miR-221-5p inhibitor-mediated osteogenic differentiation. (A) Protein expression levels of smad3 were detected by western blotting following transduction with lv-smad3 lentiviral vector. **P<0.01 vs. NC. (B) mRNA expression levels of smad3 were detected by RT-qPCR following transfection with lentiviral vector. *P<0.05 vs. NC. (C) N-mesenchymal stem cells transfected with miR-221-5p inhibitors were subsequently infected with the lentiviral vector. Images of Alizarin Red staining were captured using a light microscope. Scale bar, 100 µm. (D) RT-qPCR was performed to detect the mRNA expression levels of ALP, OPN and OC following transfection with miR-221-5p inhibitor and/or infection with lentiviral vector. *P<0.05, **P<0.01 vs. control. ^##^P<0.01 vs. lv-smad3 group. ALP, alkaline phosphatase; lv-smad3, lentiviral vector overexpressing smad3; miR-221-5p, microRNA-221-5p; NC, negative control; OC, osteocalcin; OPN, osteopontin; smad3, smad family member 3; RT-qPCR, reverse transcription-quantitative PCR.

